# Myopericarditis following both BNT162b2 and NVX-CoV2373

**DOI:** 10.1186/s13223-022-00750-7

**Published:** 2022-12-23

**Authors:** Saima Ahmad, Chino Yuson, Adrianna Le, Pravin Hissaria

**Affiliations:** 1 Royal Adelaide Hospital, Port Road Adelaide, Adelaide, SA 5000 Australia; 2 The University of Adelaide, Adelaide, Australia; 3 SA Pathology, Adelaide, Australia; 4 Present Address: Adelaide, SA 5000 Australia

**Keywords:** Myocarditis, Pericarditis, Myopericarditis, COVID-19, SARS-Cov-2, Vaccination, BNT162b2, mRNA-1273, NVX-CoV2373

## Abstract

**Background:**

Myopericarditis is a well reported complication associated with SARS-Cov-2 (COVID-19) infection and vaccinations; particularly with mRNA vaccines (BNT162b2 and mRNA-1273), and in the young male population. The risk-to-benefit ratio in sequential vaccination dosing in young males is further clouded in the era of the omicron variant with its reported enhanced immune escape.

**Study design:**

A case series of two cases of post vaccination myopericarditis following the NVX-CoV2373 after also developing myopericarditis with BNT162b2.

**Conclusion:**

To our knowledge, we are the first to describe post vaccination myopericarditis following NVX-CoV2373 after also developing myopericarditis with BNT162b2. The similarities in presentation between the reactions of both platforms would suggest a similar pathogenesis, although the exact mechanism remains unknown. Further studies are necessary to identify these mechanisms, as well as to identify biomarkers that may identify vulnerable populations. On-going vigilance is necessary to identify those who may be at an increased risk of post-COVID vaccine myopericarditis.

## Background

Myopericarditis is a well reported complication associated with SARS-Cov-2 (COVID-19) infection and vaccinations. Although, the estimated risk of developing myopericarditis post-infection with COVID-19 in the general population is substantially higher when compared with the risk post vaccination [1]. In a large case series of more than 30 million individuals in England between 1 December 2020 and 24 August 2021, the estimated risk of extra myocarditis events post vaccination was observed to be 1 to 10 per million persons [1]. The risk is highest following second dose of mRNA vaccines in young males (age under 40 years), with estimated risk of 12 excess cases per million in BNT162b2 and 101 excess cases per million with mRNA-1273, which exceeds the rate of post-infection myocarditis risk of 7 cases per million in this population group [2]. Increased risk of myocarditis and pericarditis post 2nd dose mRNA vaccination (BNT162b2 and mRNA-1273) has also been described in young males in a large national French case-series, which additionally demonstrated an increased risk of myocarditis and pericarditis post-vaccination among females under the age of 40 years [3].

The clinical course post infection and vaccination is variable with a myriad of symptoms including chest pain, dyspnoea, fatigue, and fevers [4]. The severity of cases is also diverse ranging from mild transient symptoms to reports of severe cases with fulminant cardiac failure requiring intensive care support [4]. The episodes are generally associated with changes in electrocardiogram (ECG), cardiac biomarkers and cardiac imaging [4].

The risk-to-benefit ratio in sequential vaccination dosing in young males is further clouded in the era of the omicron variant with its reported enhanced immune escape. However, recently published studies have demonstrated vaccination, particularly with booster dose, continues to provide protection from severe infection, hospitalization, and death [5]. As such, the current (April 2022) Australian recommendations for further vaccination doses, post vaccine induced myopericarditis is to defer until symptoms have completely resolved, and to consider subsequent vaccination with non-mRNA vaccines, such as NVX-CoV2373 or ChAdOx1, on a case-by-case basis [6].

Vaccine associated myocarditis is predominately reported with mRNA vaccines (BNT162b2 and mRNA-1273), however an associated risk is also described with ChAdOx1 [1]. More recently, post marketing safety data has identified 6 cases associated with NVX-CoV2373, which are not well described [7]. This case report presents 2 cases of pericarditis and myocarditis recurrence within 1 week following booster dose vaccination with NVX-CoV2373.

## Case presentation

### Case 1

A 26-year-old male with no pre-existing medical conditions or cardiovascular risk factors presented to local hospital with pleuritic chest pain and dyspnoea approximately 11 days following vaccination with 2nd dose of BNT162b2. He reported development of progressively worsening chest pain and dyspnoea 5 days post vaccination. Examination was normal with no evidence of pericardial rub or features of cardiac failure. Laboratory investigations were significant for an elevated D-dimer (1.03 mg/L) and C-Reactive Protein (CRP; 30.1 mg/L), but notable normal serial Troponin T levels (< 3 ng/L). ECG demonstrated diffuse upsloping ST elevation. Imaging with nuclear medicine ventilation-perfusion scan and CT pulmonary angiogram excluded pulmonary embolism as alternative diagnosis. The diagnosis of pericarditis was made based on Brighton Criteria [8] with level 2 certainty due to the presence of cardiac symptoms, ECG changes and exclusion of alternate diagnosis. He was discharged from emergency department with non-steroidal anti-inflammatories. He had ongoing symptoms of chest pain and dyspnoea for a total of 3 months. During this period, he was reviewed by a cardiologist and an echocardiogram was performed which was normal, and he was commenced on colchicine 0.5 mg BD for 3 months (Table 1).

**Table 1:** Summary of clinical features, laboratory, and imaging details of the two cases

Characteristics	Case 1—26 years Male		Case 2—25 years Female	
Vaccine	BNT162b2	NVX-CoV2373	BNT162b2	NVX-CoV2373
Dose no	2	3 (Booster)	2	3 (Booster)
Symptoms prior to vaccination	No	No	No	No
Onset post vaccine (days)	7	2–3	2	5
Symptoms	Pleuritic chest pain Dyspnoea	Pleuritic chest pain Dyspnoea	Chest pain Palpitations Dyspnoea Dizziness Headache Fatigue	Chest pain Palpitations Dyspnoea Dizziness
Examination	Reduced breath sounds	Normal	Normal	Normal
Troponin T/I (ng/L)	< 3	4	522	349
NT-proBNP (ng/L)	–	< 50	–	347
CRP (mg/L)	30.1	33.2	6	6.4
ECG	Diffuse upsloping ST elevation	Global ST elevation	PVCs Anterior TWI	Sinus Bradycardia (43 bpm) Anterior TWI
Echocardiogram	Normal	Normal	–	Normal
Cardiac MRI—Pre Vaccination	–	–	–	Normal (resolution of prior abnormalities)
Cardiac MRI—Post vaccination	–	–	LV and RV dilation Myocardial and pericardial oedema Trivial pericardial effusion	–
Brighton criteria level of certainty	Level 2—pericarditis	Level 2—pericarditis	Level 1—myocarditis	Level 2—myocarditis
Hospital presentation/Admission	1 day admission	Reviewed in ED	3 days admission	5 days admission
Management	Ibuprofen Colchicine	Ibuprofen Colchicine	Prednisolone Bisoprolol	Prednisolone Propranolol
Symptom duration	3 months	2 months	5 months	Ongoing

Six months after this episode of pericarditis, he received his booster vaccination with NVX-CoV2373. Prior to vaccination he was symptom free. He reports onset of similar symptoms, to the prior episode of pericarditis, with pleuritic chest pain and dyspnoea 2–3 days post NVX-CoV2373 vaccination. Examination and laboratory cardiac biomarkers were once again normal (Troponin T 4 ng/L, NT-Pro-BNP < 50 ng/L), however CRP was elevated at 33.2. ECG demonstrated global ST elevation and bedside echocardiogram was normal. He was diagnosed with pericarditis in the Emergency Department (Brighton Criteria for Pericarditis level of certainty 2) [8] and was treated with non-steroidal anti-inflammatories and recommenced on colchicine. He had persistent symptoms for approximately 2 months. Interestingly, he contracted COVID-19 infection 2 months after this second episode of pericarditis, with symptoms of a mild upper respiratory tract infection but no recurrence of the symptoms of pericarditis.

### Case 2

**Fig. 1:**
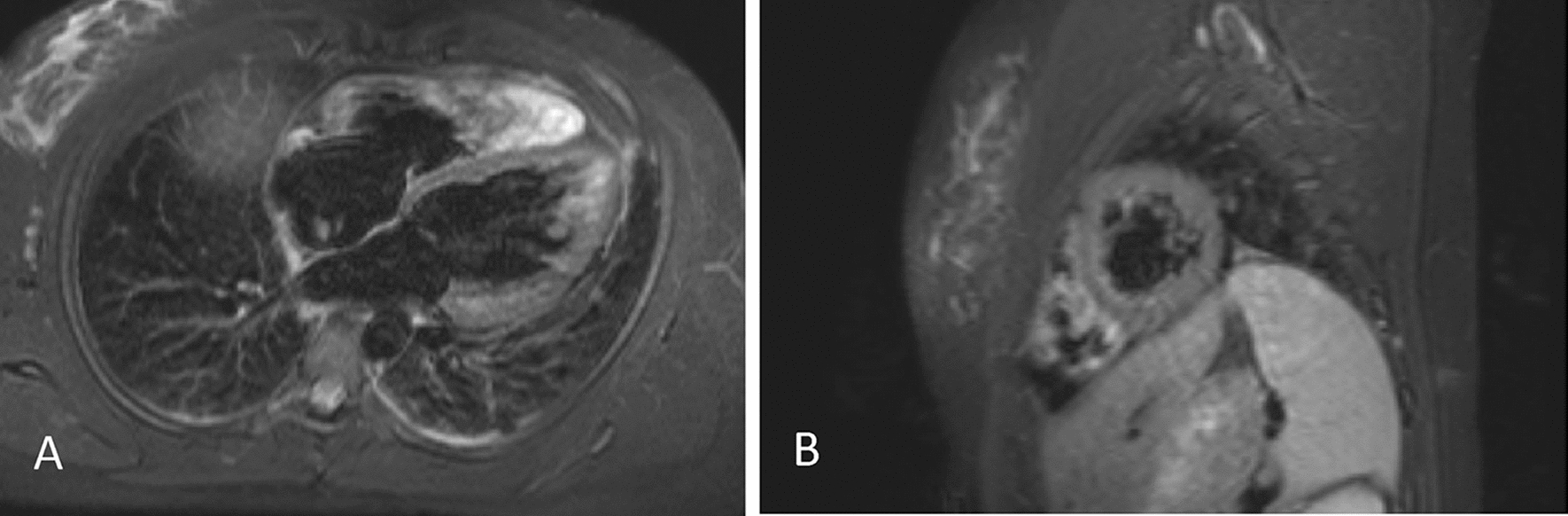
Evidence of Myocardial oedema on cardiac MRI post vaccination with BNT162b2. Transverse **A** and sagittal **B**, T2 STIR with gadolinium, images of cardiac MRI of Case 2 patient demonstrating myocardial oedema post vaccination with BNT162b2

A 25-year-old female with no cardiovascular risk factors, presented with chest pain, dyspnoea and intermittent palpitations 48 h post vaccination with 2nd dose of BNT162b2. Examination findings were normal, and ECG demonstrated frequent premature ventricular complexes (PVCs) and anterior T-wave inversion (TWI). Myopericarditis was confirmed with the presence of elevated Troponin I (522 ng/L) and abnormal cardiac MRI findings of biventricular dilation, myopericardial oedema (Fig. 1) and trivial pericardial effusion (Brighton Criteria level 1) [8]. She was commenced on regular B-blocker and tapering prednisolone regimen and had persistent symptoms requiring recurrent hospital presentations for 5 months.

**Fig. 2:**
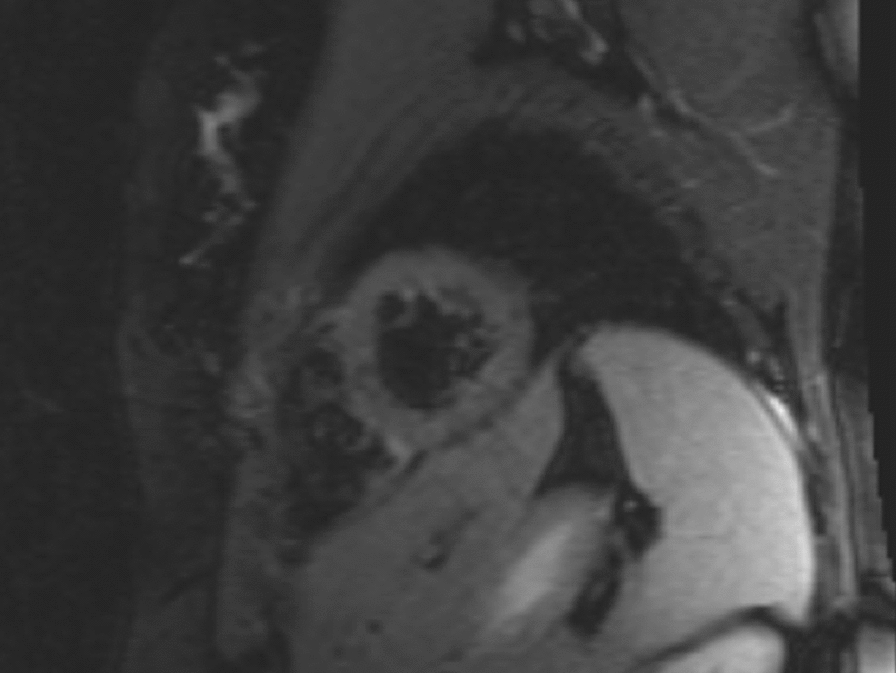
Resolution of prior oedema seen on cardiac MRI post vaccination with BNT162b2. Sagittal T2 STIR image of cardiac MRI of Case 2 patient prior to vaccination with NVX-coV2373, demonstrating complete resolution of previously observed oedema

Repeat MRI 9 months later was normal with complete resolution of the previously seen abnormalities (Fig. 2), as well as normalisation of Troponin I levels. She also remained symptom free and subsequently undertook booster vaccination with NVX-CoV2373. Five days post vaccination, she once again developed progressive severe symptoms of intermittent palpitations, dyspnoea, and chest pain. This was associated with acute elevation in cardiac biomarkers (Troponin I 349 ng/L and NT-ProBNP 347 ng/L) and a diagnosis of myocarditis was made (Brighton Criteria level 2) [8]. Echocardiogram was normal with preserved ventricular size and function. She was recommenced on tapering prednisolone regimen with subsequent gradual improvement of her symptoms. Rapid taper of prednisolone was associated with flare of symptoms and repeat hospital presentations. She reports ongoing exertional symptoms that have persisted to date at 2 months post diagnosis and vaccination.

## Discussion and conclusion

One theory that encompasses both myopericarditis following a COVID-19 infection, as well as post-COVID-19 vaccination, is molecular mimicry between the spike protein and the self-antigen, alpha-myosin, causing direct injury to the myocardium. As this hypothesis does not account for the observed skew towards mRNA vaccines, an alternative theory proposes that the immunogenicity of the mRNA strands as the cause for immune dysregulation and subsequent indirect damage to the myocardial tissue, in predisposed individuals [9].

More recently, another theory has flagged the immunogenicity of the lipid nano particle (LNP) sheath required to deliver the mRNA molecule to host cells in mRNA vaccines, as a potential cause for either direct damage to the myocardial cells, or as another trigger for immune dysregulation. Interestingly, the LNP sheath is also used in the NVX-CoV2373 vaccine, which is required for incorporation of the S-protein into the host [10].

As a shared pathomechanism would tend to lead to similar clinical presentations, it is interesting that both our subjects had mirroring events following their BNT162b2 and NVX-CoV2373 vaccinations. As young individuals who had reactions within 7 days of a non-first dose vaccination, both also fit the same demographic usually seen in post-mRNA vaccine myopericarditis. Both fulfill the criteria of myopericarditis, with presentations of pleuritic chest pain and ECG changes, although subject 1 had a mildly raised CRP and normal troponins, while subject 2 had a normal CRP with markedly high troponins.

Prior to COVID-19, post vaccine myopericarditis had been seen following smallpox vaccination. In contrast to the current COVID-19 vaccines, the smallpox vaccine is a live vaccine comprised of the vaccinia virus, itself known to cause myopericarditis. Although evidence is limited, mixed lymphocytic infiltrate with eosinophil degranulation found on one myocardial biopsy raises the possibility of a hypersensitivity or an auto-immune reaction as the potential mechanism for post-smallpox vaccination myopericarditis [11]. These findings have not been widely observed in COVID-19 vaccination myopericarditis biopsies, suggesting that a different pathogenesis may be involved [12].

Importantly, both of our subjects had clinical resolution of symptoms from their reaction following BNT162b2 for at least 3 months, with subject 2 also demonstrating radiological resolution, before again having myopericarditis after receiving NVX-CoV2373. This signifies that both subjects fit current Australian recommendations for receiving a subsequent non-mRNA vaccine following post mRNA vaccine myopericarditis.

To our knowledge, we are the first to describe post vaccination myopericarditis following the NVX-CoV2373 after also developing myopericarditis with BNT162b2. The similarities in presentation between the reactions of both platforms would suggest a similar pathogenesis, although the exact mechanism remains unknown. Further studies are necessary to identify these mechanisms, as well as to identify biomarkers that may identify vulnerable populations. On-going vigilance is necessary to identify those who may be at an increased risk of post-COVID vaccine myopericarditis.

## Data Availability

Data sharing is not applicable to this article as no datasets were generated or analysed during the current study.

## Abbreviations

CRP: C-reactive protein
ECG: Electrocardiogram
PVC: Premature ventricular complexes
TWI: T wave inversion
NT-ProBNP: NT proB-Type natriuretic peptide
LNP: Lipid nanoparticle
LV: Left ventricle
RV: Right ventricle
ED: Emergency Department
